# Judge Thayer’s Charge in the Case of Haire *vs.* Reese

**Published:** 1871-01

**Authors:** 


### *Judge Thayer's Charge in the Case of Haire* vs. *Reese.*

We quote from *The Legal Intelligencer* of November 4, 1870, the following charge of Judge Thayer, in the case of *Haire* vs. *Reese*, a full account of which appeared in our last number (see page 73). It seems to us imporant that the profession at large should be made acquainted with this document, which might serve as a model, both for skill in summing up the essential facts of the case, and for the dignified tone of consideration for the medical profession which pervades it.—*Med. Times.*

WILLIAM C. HAIRE *vs.* JOHN F. REESE, M.D.

1. The implied contract of a surgeon or a physician who attends a patient is, not that he will certainly effect a cure, but that he will use all known and reasonable means to accomplish that object, and that he will attend his patient carefully and diligently. His relation to his patient implies that he possesses, and will employ in the treatment of the case, such reasonable skill and diligence as are ordinarily exercised in his profession by thoroughly educated surgeons or physicians; and, in judging of the degree of skill which he contracts to bring to the service of his patient, regard is to be had to the advanced state of the profession at the time.
2. No presumption of the absence of proper skill and attention arises from the mere fact that the patient does not recover, or that a complete cure was not effected.
3. On the part of the patient, it is his duty to conform to the necessary prescriptions and treatment, if they be such as a surgeon or physician of ordinary skill and care would adopt or sanction; and if he will not, or, under the pressure of pain, cannot, the surgeon or physician is not responsible for injury resulting therefrom.
4. When malpractice, or want of skill or proper attention, is charged against a physician or surgeon, the burthen of proving it lies upon the person who alleges it.

GENTLEMEN OF THE JURY:

The plaintiff has brought this action against the defendant, Dr. Reese, for alleged malpractice as a physician and surgeon. The grounds upon which he alleges he is entitled to sustain this action for damages are, that the defendant treated him unskillfully for his injuries, and that he did not give that diligent care and attention to his case which it was incumbent on him to extend to him, and which he had a right to expect. That is the question which you are to determine by your verdict.

The history of the case appears, from the evidence which is before you, to be this: On the 2d of February, 1869, the plaintiff, who is by trade a house-painter, was engaged in painting the outside of the House of Refuge, in this city, when the jack upon which he was standing accidentally gave way, and he was precipitated to the ground, a distance of twenty-eight feet, his body striking violently against a fence in its fall. The defendant, who is the attendant physician of the institution, was immediately sent for, and he came promptly to his assistance. As soon as he arrived, he proceeded to take up the arteries in the head, which had been cut by the fall, and to stanch the bleeding of his wounds. When that was completed, he proceeded to examine the plaintiff's hip, in which he was suffering great pain. The plaintiff groaned with pain under the examination, and the doctor thereupon advised that he should be removed at once to his own home, where he could be better provided for, and where a more thorough examination could, with greater facility and less pain to the patient, be made. At the special request of the plaintiff he consented to attend him, and went in advance of the plaintiff to his home, in a distant part of the city, in order to prepare his family for the bad news which awaited them, and to make proper preparations for the reception of the patient. When the latter arrived, he was carried, by the doctor's directions, into the sitting-room and laid carefully upon a bed. The doctor then etherized him, in order to enable him to endure the examination to which he was about to subject him. He then proceeded to make a thorough examination of the injured parts, and, contrary to his own expectations, as he says, found, after making this critical examination, that there was neither fracture nor dislocation of the bones. After administering an anodyne to him, and directing an anodyne liniment for the hip, he left him for the night. The next day he visited him again, and made another careful examination of the hip, according to the doctor's testimony, but again found no evidences of fracture or dislocation. He prescribed liniments and anodynes, and directed him to be kept in a quiet condition. The doctor continued to visit him daily for a considerable time, making twenty-one visits in all. On the 24th of February, about three weeks after the accident, at Dr. Reese's suggestion, Dr. Agnew was called in to a consultation. He came, and made a thorough examination of the injured part. He resorted to every means known to surgery to ascertain if there had been a fracture. You will recollect the details of that examination, given to you by Dr. Agnew himself. The patient was first examined in a recumbent position. The parts were carefully manipulated and turned about; but there was no crepitation,—that is, no sound of grating of bones,—which is usually detected immediately by the practiced ear of a skilful surgeon, where a fracture has occurred. The limbs were carefully measured and compared with each other; measurements of various kinds, and in different directions, were made, to ascertain whether the injured leg had undergone any shortening. The patient was then placed in an erect posture and again examined. His leg was swung backward and forward. In short, after subjecting the limb to all the tests usually applied in such cases, Dr. Agnew, as he has testified, was convinced that there was neither fracture nor dislocation. Dr. Agnew has stated in his testimony, in view of this thorough examination to which he subjected the plaintiff, that if there then existed a fracture it could not be discovered by any human means. Dr. Reese made his last visit to the patient on the 10th of May. For the professional service which he rendered he has never received a dollar. On or about the 6th of August following, the plaintiff called upon Dr. Agnew at his office. Dr. Agnew then observed that there had been some shortening of the leg. When asked by Dr. Agnew when that shortening had commenced, he replied that "it was after he had got up to go about on crutches." Dr. Agnew advised him to get a high-heeled shoe, and to dispense with crutches. He then went away. He rewarded Dr. Agnew for his services by bringing a suit against him also. Subsequently he went to see Dr. Gross, at the Jefferson College clinic, who prescribed an ointment for his leg; whereupon the plaintiff accused him of having poisoned him. He went also to see Dr. Duffie, who advised him to throw away his crutches and to get a high heel to his shoe. While Dr. Reese was attending him, he consulted other persons without informing him of it, and applied to his leg various nostrums which they recommended. He now charges Dr. Reese with the shortening of his leg, and seeks to make him responsible for it. You are to decide whether his charge is just and true or not.

Gentlemen of the jury, before I refer to the evidence in the cause, I will direct your attention to certain principles of law which are applicable to such investigations.

The implied contract of a surgeon or a physician who attends a patient is, not that he will certainly effect a cure, but that he will use all known and reasonable means to accomplish that object, and that he will attend his patient carefully and diligently. His relation to his patient implies that he possesses, and will employ in the treatment of the case, such reasonable skill and diligence as are ordinarily exercised in his profession by thoroughly educated surgeons or physicians; and, in judging of the degree of skill which he contracts to bring to the service of his patient, regard is to be had to the advanced state of the profession at the time. The defendant in this case was bound to use reasonable skill and diligence to effect a cure; and reasonable skill and diligence means such skill and diligence as educated and faithful surgeons or physicians ordinarily employ.

No presumption of the absence of proper skill and attention arises from the mere fact that the patient does not recover, or that a complete cure is not effected. God forbid that the law should apply any rule so rigorous and unjust as that to the relations and responsibilities arising out of this noble and humane profession! The medical man who is called to attend a patient undertakes to possess such knowledge and skill as are usually and commonly possessed by educated physicians, and to apply that skill and knowledge with all due diligence and care for the benefit and advantage of the patient. If his performance comes up to that standard, he has discharged his duty and is not responsible for results. On the part of the patient, it is his duty to conform to the necessary prescriptions and treatment, if they be such as a surgeon or physician of ordinary skill and care would adopt or sanction; and if he will not, or, under the pressure of pain, cannot, the surgeon or physician is not responsible for injury resulting therefrom.

When malpractice, or want of skill or proper attention, is charged against a physician or surgeon, the burthen of proving it lies upon the person who alleges it. In the absence of satisfactory proof to establish such a charge, the presumption is that he was competent for the task which he undertook, and did his duty to the best of his ability. This is the rule of common sense, and the rule of law upon this subject. The burthen of proof, therefore, in this case, as in all similar cases, is upon the plaintiff. You are not to rush to conclusions detrimental to the reputation and interests of the defendant without competent proof. You are to decide the case by the evidence. You are sworn to give a true verdict according to the evidence. Your consciences must be satisfied by the evidence that the plaintiff's case is proved, before you can be justified in finding a verdict against the defendant. And I will add that it is your duty to weigh the evidence carefully, and to decide the cause according to the weight of the evidence.

Having thus pointed out the rules of law which are applicable to this inquiry, I will now proceed to make some references to the evidence which has been given, reminding you, at the same time, that you are the exclusive judges of the facts, and with you must ultimately rest the responsibility of deciding the cause.

The charge made by the plaintiff, as he has attempted to maintain it by the evidence, is that the defendant mistook the nature of his injury. He says that his thigh-bone was fractured, whereas the defendant assured him that it was not, and treated him as if it were not, the consequence of which was, as he alleges, the shortening of his leg. The proof upon which he relies to show that there was a fracture is, in the first place, the evidence of certain witnesses; and, in the second place, he says that the fracture is proved by the shortening itself. Now, it is apparent, from the testimony of all the surgeons who have been examined,—as well the plaintiff's as the defendant's witnesses,—that, in consequence of an injury such as the plaintiff received, shortening of the limb may result either from a fracture of the bone or from what is technically called *interstitial absorption*,—that is to say, the absorption of the extremity or neck of the femur, or thigh bone, a result frequently arising from a violent contusion. If you believe the leg was shortened, then it will be proper for you to inquire whether it was the result of an actual fracture, or of absorption taking place in consequence of a contusion; because you will observe that unless there was a fracture the plaintiff's allegation of mistake or neglect on the part of the defendant in not ascertaining that fact is not made out. Let us, therefore, examine the evidence upon this point.

The plaintiff himself says, in his testimony, very positively, that the bone was fractured. Now, it is for you to say how much weight is to be given to that statement, in view of the other testimony in the case. You will consider whether he could probably determine that point with as much certainty as the surgeons who professionally examined the limb. You will consider whether his assertion upon this point is of as much value as the testimony of the surgical experts who examined him. To me it appears a question much more difficult to be decided with certainty by the patient himself than by those who, from long experience and education, are accustomed to ascertain such facts by the scientific tests which they are accustomed to resort to in order to determine it. But the value of his testimony I leave entirely to you.

The first witness he called upon this point to sustain his own assertion was Dr. John Hirst, who is a graduate of the College of Surgeons of Edinburgh. He testified that he examined the plaintiff about two years after the accident occurred. He told him that he could do nothing for him; that he had no doubt that his case had been correctly treated, from the character of the gentleman who had attended him. He casually expressed the opinion, he says, that the leg had been fractured in the neck of the thigh-bone, and he formed that opinion, he says, from the shortening of the limb. According to his testimony, he based his opinion on that circumstance and what he had heard of the history of the case; he did not measure the limb. He further said that concussion may induce disease of the articulating head of the thigh-bone, resulting in interstitial absorption; and that will occasion a shortening of the limb. He further said that he thought the shortening was owing either to fracture or interstitial absorption. As a general rule, the limb would be very soon shortened by a fracture after the injury was received, but if shortened by absorption, the shortening would come on gradually. He also said that if he examined a patient and found that there was neither crepitation, inversion of the limb, nor shortening, he could not say there had been a fracture. Dr. Hirst does not appear, by the evidence, to have examined the plaintiff by means of the usual scientific tests described by the other surgeons. He looked at it two years after the accident, and gave a casual opinion, as he himself expresses it, founded upon a comparison, by the eye, of one leg with the other, and upon what he had heard of the history of the case. That appears to have been all the examination he gave it. It is for you to settle the weight and value of his testimony on this point.

The next witness called by the plaintiff was Moses Stevenson, who says he graduated in medicine in 1870, after studying two years. He says he examined the plaintiff's leg last winter, and believes that it had been fractured. I do not consider it worth while to dwell upon the testimony of this witness. You will recollect the exhibition which he made upon being cross-examined, saying, among other things, that "the head of the femur may be *crepitated* by absorption." In my judgment, his testimony is not worth considering, and was in the highest degree discreditable to himself. I dismiss him, therefore, without further comment.

The next witness called by the plaintiff was Dr. Joseph D. Scoles, whose testimony appeared to me to be both clear and candid. He says that he formed the opinion that the hip-bone had received an injury which occasioned the shortening of the limb; that this shortening may have been caused either by fracture or absorption, and that it is impossible for him to say which.

I have now given the substance of the whole of the plaintiff's testimony upon this subject of fracture or no fracture.

On the part of the defendant, Dr. Reese (the defendant) testified in great detail to the nature of the examination to which he had subjected the plaintiff immediately after the happening of the accident. I will not take up your time by going over it, for I am sure you will recall it. And after completing that examination, which appears, by his own statement, to have been very carefully and deliberately made, he came to the conclusion that there had been neither fracture nor dislocation.

Dr. D. H. Agnew, the distinguished Professor of Operative Surgery in the University of Pennsylvania, testified that he examined the plaintiff's limb about three weeks after the accident, and applied every test known to surgical practice to ascertain whether there had been a fracture, and was clearly of opinion that there was neither fracture nor dislocation. You will remember the description he gave you, at considerable length, of that examination, and of the various methods resorted to by him to determine the fact. He said that if there was a fracture at that time it could not be discovered by any human means. He says, moreover, after listening to the details of the treatment of the patient by Dr. Reese, that it was in all respects skilful and proper.

Dr. Gross, the eminent Professor of Surgery in Jefferson College, and a gentleman of great experience in the profession, testifies that he examined the patient at the close of a clinic, and came to the conclusion that the injury to the leg was the result of severe contusion. He further says that, if the bone had been fractured, the shortening of the limb would, beyond all question, have taken place within twelve or fifteen days after the accident. He also corroborated, to the fullest extent, the opinion of Dr. Agnew in regard to the skilful and judicious character of the treatment given to the plaintiff by Dr. Reese.

Dr. Duffie, also called as a witness by the defendant, and having also examined the plaintiff some time since, was distinctly of opinion that it was a case of absorption of the thigh-bone, a result which, he says, no remedies known to surgery can cure.

Dr. John H. Brinton, a well-known surgeon of long and large experience, testified, after hearing at length the treatment to which the plaintiff had been subjected by the defendant, that, in his opinion, it was perfectly correct and judicious, and said he knew of no other treatment for such a case.

The testimony of Dr. R. J. Levis was to the same effect. He says that the treatment was skilful and proper.

All the surgeons who were examined agree that, if there was no fracture, the treatment was perfectly proper; and they all agree that the only evidence of fracture was the shortening of the limb, and that shortening ensues with equal uniformity from absorption —the consequence of contusion—as from fracture, the only difference being that in the former case it comes on at a much later period in the history of the case than in the latter. You have then, on the one side, the positive statement of the plaintiff and the opinion of Dr. Hirst, founded upon an examination certainly not critical in its character, made two years after the accident, and upon what he had heard about the case. You have, upon the other, the opinions of Dr. Agnew, Dr. Gross, Dr. Brinton, Dr. Duffie, Dr. Packard, Dr. Levis, and Dr. Reese, the defendant.

You have also the important fact that there is no evidence that any shortening took place for a considerable period after the accident. Dr. Agnew is positive that there was no shortening when he first examined the plaintiff about three weeks after the accident, and that he did not observe that it had taken place until the plaintiff came to his office in August, about six months after the accident.

You ought to decide the case according to the weight of the evidence. If you are of opinion that the plaintiff's leg was not fractured, I do not see that there is any evidence that the case was not properly treated by Dr. Reese. I have a right to say, and I conceive it to be my duty in this case to say, that I see no satisfactory evidence that the treatment of Dr. Reese was not, in all respects, skilful, wise, humane, and proper. But I leave all the evidence to you, and you will decide for yourselves.

If, after looking over the whole case and weighing all the evidence, and applying the rules of law regulating his responsibility, to which I referred in the commencement of my charge, you conscientiously come to the conclusion that the defendant was guilty of any negligence or want of ordinary care and diligence resulting in injury to the plaintiff, of course you will not hesitate to say so by your verdict. But if, on the contrary, you come to the conclusion that the plaintiff's complaint is altogether unfounded, then it concerns not only the interests of the parties in the present cause, and not only the interests of public justice, but also the established medical fame of this city (a fame established by many examples of men great and distinguished in this profession, who have here lived and labored and died), that you put an end, so far as you can, to experiments, by unjustifiable lawsuits, against skilful, attentive, and humane physicians.*—*Med. Times.*

* It will be remembered that the jury, without leaving their box, returned a verdict for the defendant; the costs to be paid by the plaintiff.—ED.

